# Distal left circumflex coronary artery flow reserve recorded by transthoracic Doppler echocardiography: a comparison with Doppler-wire

**DOI:** 10.1186/1476-7120-5-22

**Published:** 2007-06-16

**Authors:** Antonio Auriti, Christian Pristipino, Cinzia Cianfrocca, Antonino Granatelli, Vincenzo Guido, Francesco Pelliccia, Salvatore Greco, Giuseppe Richichi, Massimo Santini

**Affiliations:** 1 Department of Cardiovascular Disease – Echocardiography Unit – S. Filippo Neri Hospital, Rome, Italy; 2 Coronary Intervention Unit and ROMA ("Ricerche Orientate sulla Malattia Aterosclerotica") core lab S. Filippo Neri Hospital, Rome, Italy

## Abstract

**Background:**

Coronary flow reserve (CFR) recording by means of transthoracic echocardiography (TTDE) in all the main distal coronary arteries is a challenge for advanced echocardiography. Validation studies of TTDE versus Doppler-wire (DW) recordings are available for Left Anterior Descending artery (LAD) and the Posterior Descending coronary artery (PD), but lacking for the more technically challenging Left Circumflex coronary artery (LCx).

**Aim:**

To evaluate the reliability of TTDE in assessing CFR in LCx when compared to the intracoronary Doppler flow-wire gold standard.

**Methods:**

we evaluated 5 patients (age = 60 ± 9 years, 5 males) on LCx by TTDE and invasive CFR assessment. TTDE recording was performed using a low-frequency probe, with a four-chamber as a guiding 2D view. The 2 tests were performed on different days and in random order within 48 hours in a blind fashion. Vasodilator stimulus was adenosine, intravenously (140 γ/kg/min × 3–6 min) for TTDE and intracoronary (40 γ bolus) for DW recordings.

**Results:**

CFR values on LCx ranged from 1.9 to 2.8 for DW, and from 2.0 to 3.0 for TTDE, with an overall correlation of R = 0,85 (p = 0,06); normal (CFR > 2.5) or abnormal (CFR < 2.5) value was concordantly identified by the 2 techniques in 4 out 5 cases (80%).

**Conclusion:**

CFR of LCx artery can be obtained noninvasively with TTDE.

## Background

Coronary flow reserve (CFR) recording by means of transthoracic echocardiography (TTDE) in all the main distal coronary arteries is a challenge for advanced echocardiography. Clinical implications of such a possibility are involving an improved indication for invasive exams and for revascularisation, the evaluation of coronary flow after revascularisation, the new generation of stress-echo testing (CFR plus wall motion) and the non-invasive follow-up of patients [1-8].

Several studies concerning coronary flow and CFR recorded in the Left Anterior Descending coronary artery (LAD) and few in the Posterior Descending coronary artery (PD) have been published so far [9-12], and the measurement of CFR by TTDE was validated, by comparing it with intracoronary Doppler recordings (DW) in the same patient and in the same vessel, in some studies [13-16].

However, only few papers concerning the distal Left Circumflex coronary artery (LCx) have been published [17-19]. Technical difficulties may pose feasibility challenges to LCx recording like the supposed lack of a reference structure to identify the position of the distal branches of LCx on the lateral left ventricle wall or the poor lateral resolution of current equipments accounted for. Moreover, a comparison of TTDE with DW for LCx is still lacking.

In the present study we compared CFR measurements obtained by TTDE with those obtained with intracoronary DW in five patients.

## Methods

### Patients population and TTDE

**Figure 1:**
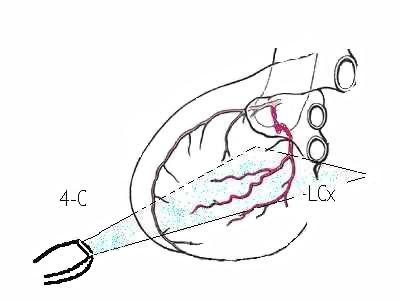
Plan of section of LCx branches made by the four-chamber view. **4-C: four-chamber view. LCx: left circumflex artery**.

**Figure 2:**
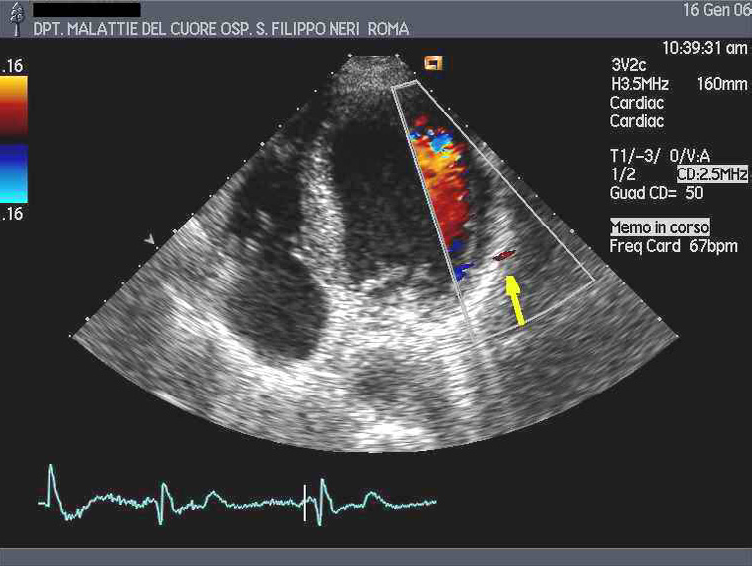
Color flow mapping of the coronary artery on the lateral wall (arrow).

**Figure 3:**
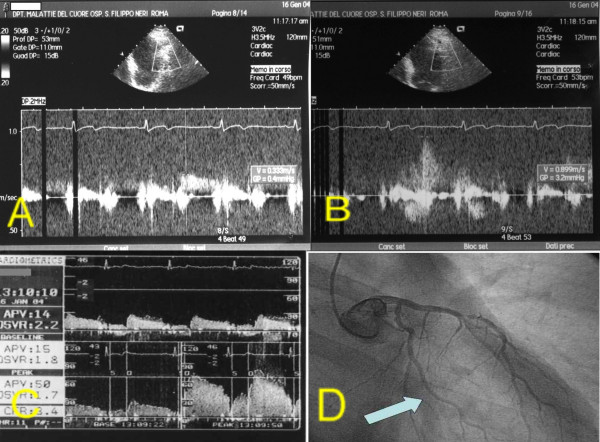
Patients 1. A: TTDE before adenosine. B: TTDE after adenosine. C: DW recording. D: coronary angiography with site of DW recording (arrow). **DW: Doppler-wire. TTDE: transthoracic Doppler echocardiography**.

**Figure 4:**
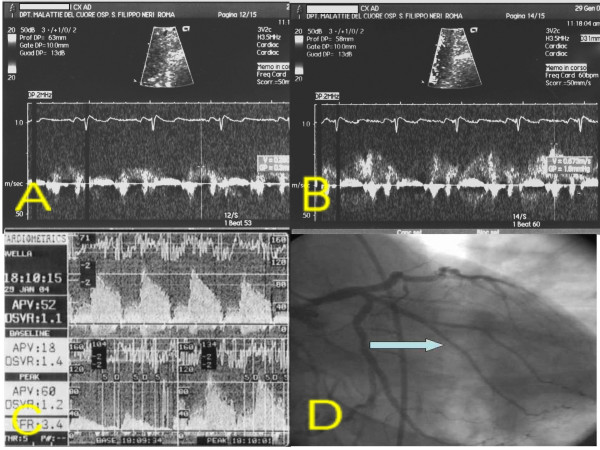
Patients 2. A: TTDE before adenosine. B: TTDE after adenosine. C: DW recording. D: coronary angiography with site of DW recording (arrow). **DW: Doppler-wire. TTDE: transthoracic Doppler echocardiography**.

**Figure 5:**
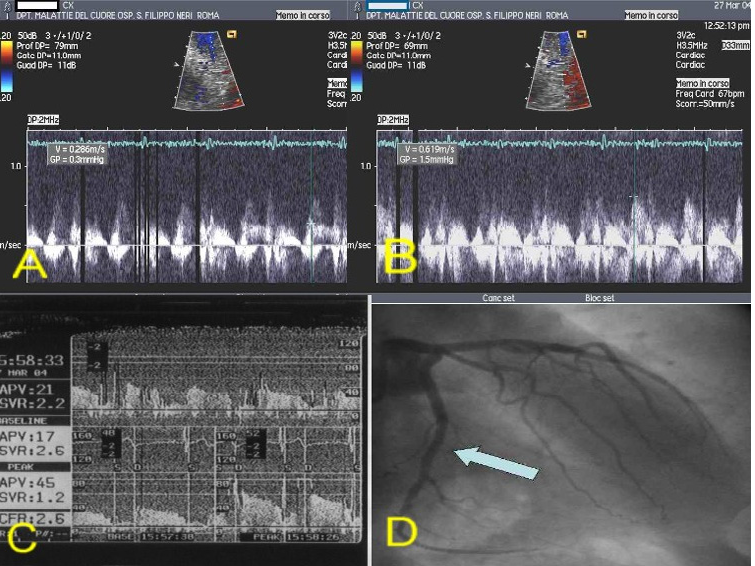
Patients 3. A: TTDE before adenosine. B: TTDE after adenosine. C: DW recording. D: coronary angiography with site of DW recording (arrow). **DW: Doppler-wire. TTDE: transthoracic Doppler echocardiography**.

**Figure 6:**
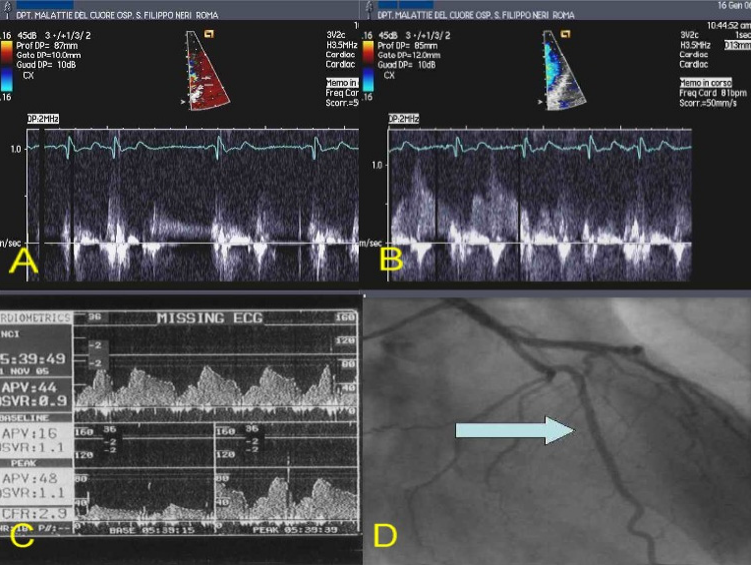
Patients 4. A: TTDE before adenosine. B: TTDE after adenosine. C: DW recording. D: coronary angiography with site of DW recording (arrow). **DW: Doppler-wire. TTDE: transthoracic Doppler echocardiography**.

**Figure 7:**
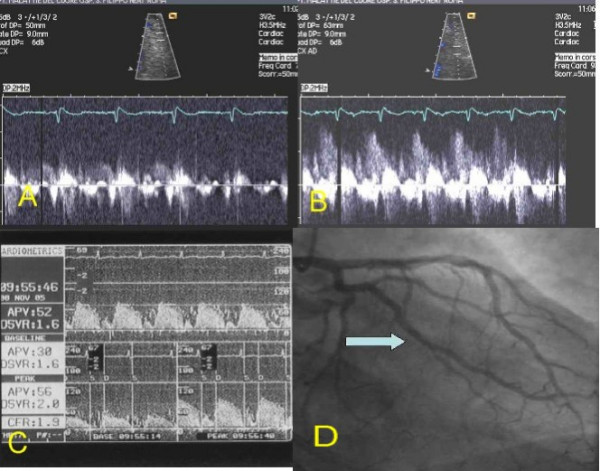
Patients 5. A: TTDE before adenosine. B: TTDE after adenosine. C: DW recording. D: coronary angiography with site of DW recording (arrow). **DW: Doppler-wire. TTDE: transthoracic Doppler echocardiography**.

Five patients (age 60 ± 9 years, 5 males, 2 with previous inferior myocardial infarction – patient 1 and 2), having a good echocardiographic window for the lateral wall, scheduled for coronary arteriography, were studied after informed consent was given. The 2 tests (TTDE and DW) were performed on different days and in random order within 48 hours in a blind fashion. Recordings of TTDE were obtained with a conventional echo-machine (Sequoia 256, Acuson-Siemens) equipped with its standard harmonic low-frequency probe (3Vc). A four-chamber view (4-C) was used for guiding TTDE recordings by adjusting frequency, limit of Nyquist (reduced to 40 cm/sec and more) and filtering of guiding color flow mapping (fig 1, 2). No contrast agent was used. Coronary flow signal search was made before by color-Doppler in the 2D view and then by pulsed Doppler. Plane of guiding 2D view was tilted and the region zoomed as necessary. The sample volume of pulsed Doppler was placed on the epicardial layer of the lateral wall in the basal and mid portions, avoiding the far apical portion where a signal recorded could belong to a Diagonal artery coming from LAD. The only reference structure relied was the lateral wall itself where Obtuse Marginal (OM) arteries (distal LCx) are running. No angle correction was used. When the typical coronary diastolic forward flow pattern along the lateral wall of the left ventricle was recorded, CFR was calculated by giving adenosine infusion (140 γg/Kg/min for 3 to 6 min) from the ratio of post-to-pre peak diastolic velocity (fig. 3A–B, 4A–B, 5A–B, 6A–B, 7A–B).

### Coronary arteriography and DW recording

After performing diagnostic coronary angiography, a 0.014" angioplasty Doppler guide wire (Flow Wire, Jomed) trough a 6 Fr guiding catheter was advanced in the target coronary branch 1 to 2 cm distal to the last stenosis (i.e. the first LCx branch with a diameter ≥ 2 mm running toward the lateral wall) or, if no angiographically detectable stenosis was present, in the proximal segment of the target branch. When a position where a clearly defined and stable velocity signal was found and recorded, 40 γ of adenosine were injected intracoronary and the maximum increase of flow velocity was recorded to calculate CFR. Adenosine injection was repeated three times and data averaged. Percent of coronary artery area stenosis was calculated by offline quantitative coronary angiography (QCA-CMS vers. 5.1, Medis, Nuenen, The Netherlands) (fig. 3C–D, 4C–D, 5C–D, 6C–D, 7C–D).

### Statistical analysis

Continuous data are presented as mean ± Standard Deviation. A sample linear regression analysis with Pearson's coefficient was used to assess the relationship between CFR values obtained with invasive (DW) and non-invasive (TTDE) methods.

## Results

CFR values ranged from 1,9 to 2,8 for invasive DW, and from 2,0 to 3,0 for TTDE, with an overall correlation of R = 0,85 (p = 0,06). A normal (CFR > 2,5) or abnormal (CFR < 2,5) value was concordantly identified by the two techniques in 4 out of 5 cases (80%) . (Table 1)

## Discussion

The non-invasive recording of the coronary flow by means of TTDE is an important progress of echocardiography in the field of coronary artery disease. The evaluation of CFR in the LAD and PD territory by TTDE already found a clinical application in the practice in some centres. Important applications are involving the status of the coronary circulation after myocardial infarction or coronary revascularisation [2,3,5,15] and the clinical use of the on-developing new generation of stress-echo [20,21]. As stated, from a physiological point of view, the largest part of informations comes from recording the flow from a distal segment of the coronary artery. This explains why CFR measured by transesophageal echocardiography on the proximal coronary arteries does not correspond to the status of the distal coronary circulation.

LCx distal CFR recording has been reported until now in few papers only [17-19] with a described feasibility of about 80%; however, a comparison with DW method is still lacking.

In the present paper we recorded the flow reserve from the arteries running along the basal and mid lateral wall. These arteries are the OM branches of LCx if the recording is made on the basal or mid lateral wall in a 4-C plane. In fact, the plane of the Diagonal branches of LAD is lying superiorly (fig 1). No reference structure other than the lateral wall itself is necessary to be followed to record the distal LCx, in our opinion. Following these rules, in our patients, we could observe a good comparability in CFR measurements between TTDE and DW either in terms of profile of the Doppler spectrum and in the values of CFR. The borderline statistical significance ought to be due to the sample tininess.

We underline that the present study was neither a feasibility one nor it was its target to assess the improvement of feasibility by using contrast agents. This is why we enrolled non-consecutive patients. To add informations around CFR, particularly in the region of the lateral wall, would be very helpful in assessing the presence of global ischemic response to vasodilators in the course of stress test, as long as this region has a low sensitivity for wall motion marker alone. However, further large studies are needed to precisely assess the applicability of LCx CFR calculation by TTDE in the clinical practice in order to completely evaluate, through a non invasive method, the functional status of coronary circulation.

## Limitations

A problem raised in TTDE flow recordings of distal LCx is that one is not certain about which of the vessel on the lateral wall (OM1, OM2) is recorded. However, this is the same for LAD recordings when sometimes a Diagonal branch is recorded in the position of distal LAD. And, in any case, a result of an impaired CFR might be a clinically relevant pushing drive for a further work-up.

For technical reasons only few patients were studied here and larger series are necessary to substantiate our pilot observation.

## Abbreviations

**Vmax1 **= flow velocity before Adenosine (m/s)

**Vmax 2 **= flow velocity after Adenosine (m/s)

**CFR **= coronary flow reserve

**TTDE **= transthoracic Doppler echocardiography

**Pt **= patient

**LCx **= Left Circumflex artery

**OM **= Obtuse Marginal artery

## Authors' contributions

All Authors have read and approved the final manuscript.

**Table 1:** Results

	**TTDE**			**Doppler-wire**			**Quantitative angio**	
	**Vmax1**	**Vmax2**	**CFR**	**Vmax1**	**Vmax2**	**CFR**	**% area stenosis**	**Vessel**

**Pt 1**	0,30	0,9	**3**	0,3	0,7	**2,3**	**0**	**OM3**
**Pt 2**	0,30	0,7	**2,3**	0,3	0,6	**2**	**37**	**OM1**
**Pt 3**	0,3	0,6	**2**	0,2	0,4	**2**	**70**	****dist LCx****
**Pt 4**	0,3	0,9	**3**	0,28	0,77	**2,8**	**0**	**OM1**
**Pt 5**	0,5	1	**2**	0,43	0,8	**1,9**	**13**	**OM1**
